# Circadian disruption and breast cancer: An epigenetic link?

**DOI:** 10.18632/oncotarget.4343

**Published:** 2015-07-09

**Authors:** David Z. Kochan, Olga Kovalchuk

**Affiliations:** ^1^ Department of Biological Sciences, University of Lethbridge, Lethbridge, AB, Canada

**Keywords:** breast cancer, circadian disruption (CD), epigenetics, circadian rhythms, melatonin (MLT)

## Abstract

Breast cancer is already the most common malignancy affecting women worldwide, and evidence is mounting that breast cancer induced by circadian disruption (CD) is a warranted concern. Numerous studies have investigated various aspects of the circadian clock in relation to breast cancer, and evidence from these studies indicates that melatonin and the core clock genes can play a crucial role in breast cancer development. Even though epigenetics has been increasingly recognized as a key player in the etiology of breast cancer and linked to circadian rhythms, and there is evidence of overlap between epigenetic deregulation and breast cancer induced by circadian disruption, only a handful of studies have directly investigated the role of epigenetics in CD-induced breast cancer. This review explores the circadian clock and breast cancer, and the growing role of epigenetics in breast cancer development and circadian rhythms. We also summarize the current knowledge and next steps for the investigation of the epigenetic link in CD-induced breast cancer.

## INTRODUCTION

Breast cancer is currently the most common malignancy affecting women worldwide. It accounts for 25% of all cancers in women and caused 522,000 deaths worldwide in 2012 [1]. Breast cancer is also present in human males, with males having poorer outcomes due to delays in diagnosis and estimates by the American Cancer Society indicating that there will be around 2350 new cases of male invasive breast cancer in the United States in 2015 [2, 3]. The development of breast cancer emerges through a multi-step process, encompassing progressive changes from a normal cell to hyperplasia, carcinoma *in situ*, invasive carcinoma, and metastasis, with the cancer either originating in the milk ducts, milk-producing lobules, or connective tissues [4–6]. Breast cancer tumours also vary in gene expression patterns and other characteristics, resulting in different classifications and treatment options.

Currently, breast cancers are separated into two main classes and four groups based on specific phenotypes. The two main classes are dependent on the hormone status of the cancer, with estrogen receptor (ER) and progesterone receptor (PR) positive cancers being part of the luminal class, and the ER and PR negative cancers being distinguished as non-luminal [7]. The luminal status of the breast cancer is then combined with the expression status of the erb-b2 receptor tyrosine kinase (*ERBB2*) gene, a proto-oncogene which encodes for the human epidermal growth factor receptor 2 (HER2) protein, in order to determine if an overabundance of HER2 plays a role in tumour progression and to classify the cancer into one of four specific groups: luminal A (group 1), luminal B (group 2), HER-2 positive (group 3), and triple negative (group 4) [7]. Groups 1 and 2 are the hormone positive breast cancers, with luminal A being HER2 negative and luminal B being HER2 positive. Groups 3 and 4 are the non-luminal cancers, and as the group names suggest, the HER2-positive group only expresses HER2 and the triple negative group is negative for all three of the phenotypes tested.

Based on the four-group classification system discussed, breast cancer patient treatment options have become more specific and efficient, and the prognosis of patients has become better and more accurate. In terms of the luminal breast cancers, these tumours are less aggressive, lead to a better prognosis, and respond to more treatment options. For example, Tamoxifen (TAM), an ER antagonist drug, is the current treatment choice for groups 1 and 2 at any stage in pre- and post-menopausal women [8, 9]. However, not only is TAM treatment not a valid option for groups 3 and 4 because there is no ER expression in these tumours, but non-luminal breast cancers are more aggressive, offer a poorer prognosis, and respond to fewer treatment options [8, 10]. As a result, a potential approach to treating non-luminal breast cancers is to reactivate ER activity through drugs such as 5-aza-2`deoxycytidine (5-aza) or Trichostatin A (TSA), and then apply TAM treatment or chemotherapy [8, 11, 12]. Although this approach may not be as efficient and proven as regular hormonal treatment, it represents a step in the right direction that was made possible by investigating the characteristics of different breast cancers.

In 2007, the International Agency for Research on Cancer (IARC) concluded “shift work that involves circadian disruption is probably carcinogenic to humans” [13]. Although this statement refers to shift work as a potential carcinogen, shift work is not the only potential cause of circadian disruption (CD). Time zone changes, jet lag, space travel, psychiatric disorders, and even light exposure from lamps and electronics during the evening hours can cause circadian disruption [14–18]. Furthermore, studies have shown that CD can promote the spontaneous development of a variety of tumours in a rodent model, and that CD may be linked to a higher risk of prostate cancer in men [19–21]. However, the most prominent link established thus far between CD and carcinogenesis has been in breast cancer, and recent case studies have supported the 2007 IARC claim by providing indirect evidence that shift workers are at a higher risk of developing breast cancer. Specifically, studies found that work after midnight significantly increased the risk of breast cancer in women when compared to day work, that the risk increased with the duration and accumulation of overnight shifts, and that rotating shifts between day and night is more disruptive than permanent night work [13, 22]. These case study findings, in combination with the fact that one third of the Canadian labour force is not working a regular daytime shift and that shift work may cause a higher prevalence of ER negative breast cancer, warrants further investigation into the mechanisms underlying breast carcinogenesis due to circadian disruption [23, 24]. By investigating the characteristics and mechanisms involved in CD-induced breast cancer, valuable insights and knowledge may materialize, thus potentially allowing for different treatment options to emerge and improving the prognosis of patients.

## CIRCADIAN RHYTHMS

A circadian rhythm is any biological process that displays an endogenous, entrainable oscillation of approximately 24 hours, with these biological processes being controlled by a circadian system that is organized in a hierarchical manner. Acting as a link between the nervous and endocrine systems via the pituitary gland and containing a number of small nuclei, including the suprachiasmatic nucleus (SCN), the hypothalamus of the brain represents the top of a circadian hierarchy through the SCN [25, 26]. Guided by the activity of the SCN, numerous hormones and genes are entrained to 24-hour rhythms of expression and activity to ensure proper cellular function [26]. Amongst these circadian-regulated hormones is melatonin (MLT), a pineal hormone that has been shown to fluctuate based on environmental light and play a crucial role in breast cancer development [26, 27]. The core clock genes are among the circadian-regulated genes. Found in almost all nucleated cells of the human body, the core clock genes not only help maintain circadian rhythm activity in peripheral oscillators but may also play a significant role in breast cancer development [26, 27].

### Suprachiasmatic nucleus

The paired suprachiasmatic nucleus of the anterior hypothalamus acts as a key oscillator of circadian rhythms by regulating downstream peripheral oscillators via endocrine and neural signals [28]. This “master” oscillator is composed of two distinct regions, a shell and a core [29]. The shell region exhibits uniform oscillations of activity, is not immediately responsive to photic stimulation, and can maintain an approximate 24 hour pattern of activity in the absence of environmental stimuli [30, 31]. Unlike the shell, the core does not display synchronized, rhythmic patterns of activity in the absence of an external input [32]. Instead, the core is externally synchronized by environmental light through non-visual, photic retinal ganglion cells [27, 30, 33]. Working together, the core interprets and analyzes information regarding the time of day and entrains the oscillator present within the shell [29]. The shell then regulates the output of the SCN, resulting in the organization of bodily processes on an environmentally synchronized schedule for optimal performance [29, 31].

In humans, many biochemical processes, such as hormone secretion, cell cycle, apoptosis, and gene expression, are entrained on a regular 24-hour rhythm by daily periods of light exposure. Repeated unnatural exposure to light during the dark phase of the circadian cycle, such as through shiftwork, disrupts the activity of the SCN and inhibits synchronizing of the circadian clock to the proper light-dark cycle [34]. Although the SCN has the capacity to adjust to new schedules, this adjustment does not occur instantly; instead, it occurs over a certain number of 24-hour cycles [27]. Evidence also shows that the majority of shift workers never actually entrain fully to the aberrant light-dark schedule, resulting in continuously desynchronized circadian rhythms [35, 36]. Furthermore, peripheral oscillators depend on the SCN for guidance. However, the SCN adapts faster than peripheral oscillators to environmental stimuli, and as a result, there is a lag that occurs which causes a desynchronizing between the SCN and the peripheral oscillators [26]. During this lag period, a multitude of rhythmic events can become disrupted, resulting in circadian phase shifts that can lead to complications and disease [26].

### Melatonin

Melatonin (MLT), a pineal hormone that plays a role in the control of sleep, is directly linked to circadian rhythm activity through the suprachiasmatic nucleus. As a result of this integration, not only does the concentration of MLT fluctuate during a circadian cycle, but the ability of melatonin to influence the SCN also changes [37]. Through a biological timing signal that is internally driven by the central pacemaker in the SCN, MLT is very abundant during the night and almost completely absent during the day, with sleep not being necessary to initiate the increase in melatonin levels but complete darkness being an absolute requirement [38, 39]. Therefore, aberrant exposure to light at nighttime can result in lower melatonin levels and potential complications.

As mentioned previously, TAM is the current treatment choice for ER-positive breast cancers [8, 9]. However, a recent study utilized a rodent model to provide evidence that dim light at night can induce resistance to TAM treatment in ER-positive tumours [40]. The resistance seems to be linked to lower nocturnal levels of melatonin because rats that did not have a disrupted MLT rhythm or rats that received nocturnal MLT replacement still responded to TAM treatment [40]. These results are striking because evidence has already emerged that shift work may result in a higher incident of ER negative breast cancer, thus indicating that CD-induced breast cancer may pose a very unique and serious problem in terms of treatment options [24]. Interestingly, the study also showed that endogenous circadian MLT increases tumour sensitivity to TAM, which is consistent with previous studies that illustrated the same findings [40–42]. These results add to the growing evidence for the potential of chronotherapy in cancer treatment. Human clinical trials utilizing a chronotherapy approach in the treatment of acute lymphoblastic leukemia and metastatic colorectal cancer have provided significant evidence of increased survival rates depending on the time of treatment within a 24-hour day [43]. In terms of breast cancer chronotherapy, a phase I human trial has provided preliminary evidence that chronomodulated infusion of a combination of chemotherapy drugs can result in acceptable toxicity, and a rodent model study has provided evidence for an optimal celecoxib dosing time based on based on the light-dark cycle [44, 45]. These findings, coupled with the fact that TAM activity is linked to circadian rhythms through MLT, provides a strong argument that chronotherapy is a logical and promising endeavour that needs to be explored in the treatment of breast cancer.

Evidence shows that melatonin appears to be involved in cancer development and growth. In experimental rat models of chemical carcinogenesis, the physiological melatonin signal suppresses the initiation phase of tumourigenesis by suppressing the damage and alterations caused by DNA adducts [39, 46]. This ability of melatonin to suppress DNA adduct damage is believed to be due to melatonin acting as a potent free-radical scavenger and/or promoting repair of DNA once damage has occurred [39, 46]. Studies also show that at nocturnal concentrations, melatonin may exhibit biochemical and molecular oncostatic actions. In a rat MCF-7 breast cancer cell xenograft study, evidence showed that constant exposure to light resulted in deregulation of the circadian MLT rhythm, and that the tumour xenografts associated with constant light exposure showed significantly increased growth when compared to the light-dark groups [47]. Another study, utilizing DMBA induced mammary adenocarcinomas in female rats, illustrated that a constant dim light during the dark phase of the circadian cycle caused higher rates of tumour growth and lower levels of survival [48].

A possible explanation for the nocturnal, light-induced tumour growth may be that lower levels of MLT cause increased metabolism of fatty acids and aberrations in cancer-dependent pathways. For example, melatonin can cause receptor-mediated inhibition of cyclic adenosine monophosphate (cAMP), resulting in downregulation of the transcriptional expression of ERα in human breast cancer cells and lower fatty acid metabolism by tumour cells through decreased fatty acid transport [39, 47, 49]. Calmodulin (CaM) activity, which plays a role in breast cancer tumour development by suppressing apoptosis and increasing survival through EGF-initiated activation of protein kinase B (Akt), is also lowered by melatonin activity through binding of the pineal hormone to CaM and by melatonin stimulating phosphorylation of CaM through protein kinase C α (PKCα) [50–52]. Further studies have also shown that human breast cancer xenografts exhibit a day-night rhythm of tumour proliferation, fatty acid uptake, metabolism, and signal transduction activity, all believed to be driven by the nocturnal, circadian rhythm melatonin signal [39, 46].

### Clock genes

**Figure 1:**
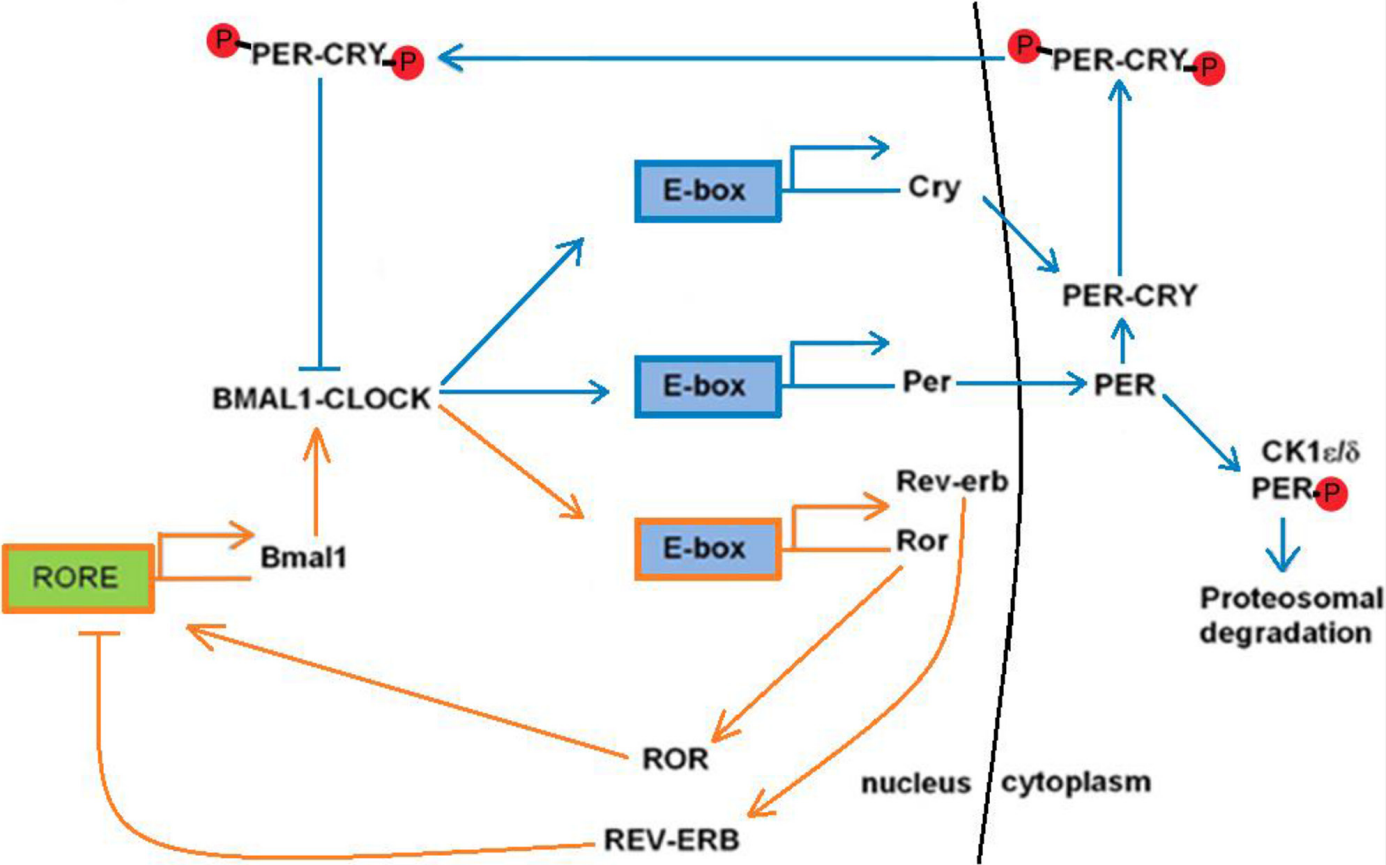
Overview of the two interlocking circadian clock regulatory feedback loops The blue arrows represent the BMAL1/CLOCK regulatory feedback loop which controls the expression of the *Per* and *Cry* genes. The orange arrows represent the regulatory feedback loop that controls the expression of the *Bmal1* gene and formation of the BMAL1/CLOCK heterodimer.

In mammals, two to ten percent of all gene expression is rhythmic, and these clock genes, located in peripheral oscillators and found in almost all nucleated cells of the human body, regulate practically every biological process and function in a time-specific manner to help maintain circadian rhythms [28, 53]. Aiding in the maintenance of the circadian clock, the core clock genes function through two interlocking regulatory feedback loops driven by the genes *CLOCK/Npas2*, *Bmal1*, *Period* (*Per*) and *Cryptochrome* (*Cry)* [54] (Figure 1). In one regulatory loop, two transcriptional activators, BMAL1 (brain and muscle ARNT-like protein 1) and CLOCK (circadian locomotor output cycles kaput), form CLOCK/BMAL1 heterodimers in the nucleus where they activate the expression of the *Per* and *Cry* genes by binding to E-box promoter sequences (Figure 1). In the cytoplasm, the PER and CRY proteins form complexes with each other, move into the nucleus, and inhibit the activity of the CLOCK/BMAL1 heterodimers, thus causing transcription of the *Per* and *Cry* genes to stop (Figure 1). In the other regulatory loop, the expression of the *Bmal1* gene is controlled through CLOCK/BMAL1 heterodimer formation in the nucleus (Figure 1). These heterodimers bind to the E-box promoter sequences of genes that encode the retinoic acid-related orphan nuclear receptors (ROR), Rev-erbα and Roraα; The Rev-erbα and Roraα proteins then compete for the ROR element (RORE) in the *Bmal1* promoter and suppress or activate *Bmal1* expression, respectively (Figure 1).

Through utilization of these circadian feedback loops, the precision of the mammalian clock is controlled through post-translational modifications [55]. Casein kinase one epsilon (CKIε) and casein kinase one delta (CKIδ), are the two main kinases responsible for the phosphorylation of the PER and CRY proteins [28]. Through the activity of these kinases, by the end of a regular circadian cycle, the PER1 and PER2 proteins have almost entirely undergone degradation, thereby preventing CLOCK/BMAL1 suppression in the nucleus and initiating the start of the next circadian cycle [56]. Nuclear entry of the PER and CRY proteins also acts as a vital checkpoint for progression of the circadian cycle, with a co-dependency existing between the two proteins [28]. The presence of CRY1 and PER2 interaction in the cytoplasm results in PER2 stability by preventing its degradation through CKIε phosphorylation, and thus permits nuclear translocation and suppression of the CLOCK/BMAL1 heterodimer (Figure 1). Alterations to the balance between the CRY and PER proteins can therefore lead to changes in the circadian cycle. For example, the *Tau* mutation in the *CKIε* gene leads to altered levels of phosphorylation of PER proteins, resulting in a shortened circadian length [57]. Furthermore, *Per1* and *Per2* respond and adapt faster than *Cry1* to circadian disruption, and as a result, circadian disruption can induce internal phase shifts within the circadian clock [58].

### Clock genes and breast cancer

In addition to helping regulate circadian oscillations, the core clock genes have been shown to influence cancer related mechanisms such as cell proliferation, apoptosis, cell cycle, and tumour-suppressor genes. Studies have shown that overexpression of *Per1* and *Per2* inhibits the growth of various cancer cells. *Period1* plays a role in apoptosis in human cancer cells, with its down-regulation stopping apoptosis and its overexpression increasing DNA damage-induced apoptosis [59]. The *Per2* gene has been shown to act as a tumour suppressor in luminal breast cancers by linking the circadian oscillator system to the ERα function [60]. Human *Per2* is estradiol (E2) inducible in mammary cells, causing *Per2* expression to lower ERα activation through the consumption of E2 [27]. Since ER activation through E2 results in DNA adduct formation, mammary cell proliferation, and genotoxic waste, the reduction in ERα activation by *Per2* expression results in an oncostatic effect [61].

The overexpression of CLOCK has been linked to increased proliferation in luminal breast cancer cells [62]. This oncogenic influence of CLOCK is likely associated with estrogen-estrogen receptor α (E2- ERα) signaling, with E2 promoting the binding of ERα to estrogen response elements (EREs) of the *CLOCK* promoter and increasing transcription [62]. Numerous single nucleotide polymorphisms (SNPs) in the *CLOCK* gene have also been correlated with increased breast cancer risk [63]. Interestingly, 67% of the SNPs were associated with non-luminal breast cancers, indicating that *CLOCK* expression most likely plays a role in both luminal and non-luminal breast cancer [63]. The same study also showed that CLOCK knockdown results in increased expression of various tumour suppressor genes and decreased expression of multiple oncogenes, indicating an oncogenic influence by CLOCK on breast cancer development [63].

Unlike CLOCK, BMAL1 has been reported as having oncostatic effects in cancer development. Specifically, BMAL1 has been shown to suppress cancer cell invasion by antagonizing the oncogene B-cell lymphoma w (*Bcl-w*), thus suppressing matrix metalloproteinase-2 (MMP-2) accumulation and blocking the PI3K-AKT-MMP2 signaling pathway [64]. Furthermore, loss of *Bmal1* expression has been shown to result in lower transcript levels of *Per1* and *Per2*, and increased protein levels of cyclin D1 [65]. The cyclin D1 protein is involved in promoting transition from the G_1_ to the S-phase in the cell-cycle by binding to cyclin-dependent kinase 4 (CDK4) or CDK6 proteins, and its overexpression enhances the activity of ERα and counteracts the breast cancer-suppressor gene breast cancer 1, early onset (*BRCA1*) [66]. In mice and cancer patients, overexpression of Cyclin D1 is associated with mammary tumourigenesis and poor medical prognosis, respectively [27, 67].

As an extension of the influences of CLOCK and Bmal1 on cancer development, the CLOCK/BMAL1 heterodimer has been shown to regulate cell-cycle genes that are involved in breast cancer progression [27]. This regulation occurs in an oncogenic manner through the activation of WEE1 G2 checkpoint kinase (WEE1), and an oncostatic manner through the suppression of myc avian myelocytomatosis viral oncogene homolog (c-MYC) [27, 67–69]. In breast cancer cells, the G_1_ cycle checkpoint is often aberrantly regulated, allowing cells with DNA damage to enter the S phase [70]. When this occurs, cancer cells rely on the G_2_ check point for damage repair before entering mitosis [70]. Over expressed in breast cancer cells, the WEE1 protein is a nuclear kinase that helps regulate transition from the G_2_ phase to mitosis by inhibiting the activity of the CDK1 protein, thus increasing DNA repair in cancer cells prior to mitosis [70, 71]. *c-Myc* is a regulator gene whose overexpression causes increased cell proliferation through cyclin activity and lowered apoptosis through Bcl-2 activity, with *c-Myc* overexpression eventually leading to hyperplasia and tumour development [67].

Although both the *Cry* genes may be implicated in breast cancer development, the majority of findings thus far have pinpointed *Cry2* as playing a more prominent role. In a study investigating the potential significance of *Cry2* in breast cancer development, experimenters identified SNPs in the *Cry2* gene as potential biomarkers for increased risk of breast cancer, with the variants having a stronger association with non-luminal breast cancers [72]. The results also illustrated that the degree of *Cry2* expression can vary between breast cancer types, with the non-luminal breast cancers having higher levels of *Cry2* expression when compared to luminal breast cancers [72]. Finally, CRY2 knockdown in MCF-7 cancer cells resulted in aberrant regulation of cell differentiation, proliferation, motility, angiogenesis, and apoptosis, thus suggesting a tumour-suppressor role for CRY2 in breast cancer development [72].

As discussed thus far, there is clear evidence connecting the circadian clock and its components to breast cancer development through hormone activity and the influence of core clock genes. However, there is another area of study that may provide the crucial link in CD-induced breast cancer.

## EPIGENETICS

Epigenetics is the study of heritable changes in gene expression or cellular phenotype by mechanisms that do not involve direct changes to the DNA sequence. Two examples of epigenetic mechanisms are DNA methylation and RNA associated silencing.

### DNA methylation

The biochemical process of DNA methylation involves the addition of a methyl group to the 5′ carbon of the cytosine nucleotide in DNA, with cytosine DNA methylation being one of the most widely studied and known epigenetic mechanisms [73]. This process plays a crucial role in normal cell development, cell proliferation, and maintenance of genome stability [73]. In mammals, DNA methylation is mediated by DNA methyltransferase enzymes. DNA methyltransferase 1 (DNMT1) is the major enzyme involved in maintenance DNA methylation and is responsible for copying DNA methylation patterns to daughter strands during DNA replication [74]. DNA methyltransferase 3a (DNMT3a) and DNMT3b act as de novo methyltransferases that target unmethylated and hemimethylated sites, and are responsible for DNA methylation patterns early in development [75]. Finally, although DNMT3L has no methyltransferase activity, it interacts with DNMT3a and 3b to help establish maternal methylation imprints and appropriate expression of maternally imprinted genes [76].

The activity of DNA methyltransferases mostly occurs in the context of CG dinucleotides (CpG), with about 60–80% of CpG cytosines being methylated in mammals [77]. Hypermethylation of CpG dinucleotides, which is over methylation of DNA, is linked to genomic instability due to silencing of DNA repair genes, silencing of tumour-suppressor genes, and compaction of chromatin [78]. Hypomethylation of CpG dinucleotides, which is the absence of cytosine methylation, has been linked to gene reactivation, chromosomal instability, and up-regulation of proto-oncogenes [79]. The ability of DNA methylation to cause transcriptional repression is achieved through methyl CpG-binding domain (MBD) proteins, with these proteins selectively interacting with methylated DNA in order to achieve gene silencing [73]. Many genes in mammals also have CpG islands that are found close to promoter regions of genes [80]. These short stretches of DNA, located in the 5`-regions of 60% of all genes, contain high concentrations of CpGs, with the methylation status of these islands usually being reversed in tumour cells when compared to normal cells for genes that play a role in cancer development [78].

### DNA methylation and breast cancer

In recent years, recognition of the role DNA methylation plays in the etiology of breast cancer has increased. In terms of hypermethylation, more than 100 genes have been reported to be hypermethylated in breast tumours or breast cancer cell lines, with many of these genes playing a role in DNA repair, cell-cycle regulation, apoptosis, and metastasis. Among the hypermethylated genes involved in DNA repair are *BRCA1* and RAD9 Homolog A (*RAD9*) [78]. *BRCA1* is an extensively studied tumour-suppressor gene involved in double-stranded DNA breaks through homologous recombination repair in late S and G_2_ phases of the cell-cycle, while RAD9 is involved in base excision repair, mismatch repair, and homologous recombination repair [81, 82]. Some of the hypermethylated genes involved in cell-cycle regulation are dickkopf WNT signaling pathway inhibitor 3 (*DKK3*) and wnt inhibitory factor 1 (WIF1), and some of the genes involved in apoptosis are homeobox A5 (*HOXA5*) and target of methylation-induced silencing (*TMS*1) [78, 83]. Both *WIF1* and *DKK3* are Wnt signaling-pathway antagonists, with increased Wnt pathway activity being associated with lowered cell senescence, resistance to apoptosis, and increased resistance to anti-cancer therapies [83, 84]. *HOXA5* expression increases apoptotic activity in a p53-dependent manner and through caspase activity [85]. Although the exact mechanism by which *TMS1* induces apoptosis has not yet been fully elucidated, *TMS1* is a proapoptotic gene and a sensitive prognostic marker for tumourigenesis [86]. Hypermethylation of tumour-suppressor genes can also be an early marker in breast cancer and be compounded by DNMT activity. For example, hypermethylation of the tumour-suppressor gene Ras association domain-containing protein 1 (*RASSF1A*) is an early epigenetic event in breast cancer, and it has been shown to increase linearly in benign breast tissue of women between 23 and 55 years of age [87].

The hypermethylation status of certain genes has also been linked as a predictor of hormone receptor development in breast carcinogenesis. DNA methylation of estrogen receptor 1 *(ESR1)* and progesterone receptor *(PR)* promoters has been proposed as a mechanism for the development of ER-negative tumours [88–90]. A recent clinical study has also shown that ERα-promoter hypermethylation has a strong correlation with ER-negative tumours, PR-negative tumours, and non-luminal tumours [91]. Furthermore, another study conducted a CpG methylation-pattern analysis on known breast cancer tumour-suppressor genes in order to correlate methylation status with hormone expression. The study found that a combination of reversion-induced LIM (*RIL*) and cadherin 13 (*CDH13*) hypermethylation was strongly correlated with ER- and PR-negative tumours, while a combination of high in normal (*HIN-1*) and *RASSFIA* methylation was associated with ER- and PR-positive tumours [92].

Although DNA hypomethylation was the first epigenetic abnormality discovered in human tumours, it was somewhat cast into the shadows at the expense of tumour-suppressor genes and hypermethylation [93, 94]. More widely spread across the genome and associated with repetitive DNA elements, DNA hypomethylation was not only ignored, but it was more difficult to pinpoint with limited technology [93, 95]. However, as scientific technologies have continued to improve, the role of DNA hypomethylation in breast cancer development has become more illuminated.

Among the types of repetitive DNA elements that are affected by global hypomethylation are transposable DNA elements [96]. Constituting 11% and 17% of the human genome, long interspersed nucleotide element-1 (LINE-1) and Alu are major components of these repetitive transposable elements [97]. Given their vast distribution in the genome, these regions are often used as markers for genome-wide methylation status [98]. In addition, hypomethylation of these regions has been linked to various cancers, with lower methylation causing transcriptional activation, retrotransposition, genomic instability, and the decrease in methylation correlating with cancer progression [98, 99]. In a recent study, experimenters investigated the link between Alu and LINE-1 hypomethylation and breast cancer subtypes [98]. The results showed that Alu hypomethylation was correlated with an ER-negative status in invasive breast cancer (IBC), while LINE-1 hypomethylation was correlated with an ER-negative status and HER2 expression [98]. The findings also suggested that LINE-1 hypomethylation is an early event in breast cancer development, while Alu hypomethylation is a late event in breast cancer progression, and that lower Alu methylation is associated with poorer disease-free patient survival [98].

Among the advances in technology that have allowed for further insight into DNA hypomethylation and cancer research is whole-genome shotgun bisulfite sequencing (WGSBS). Through the use of this technique, researchers have identified that cystosines methylated in the CpG content are almost completely methylated in pluripotent cells, but are only partially methylated in somatic cells [100]. Regions of these partially methylated cystosines can be found in nearly 40% of the genome and have been classified as partially methylated domains (PMDs) [100]. Although hypomethylation has been traditionally associated with increased gene expression, a recent study utilized WGSBS on HER2-positive breast cancer cells and found that global hypomethylation is associated with PMDs and gene silencing [101]. Specifically, a significant fraction of genes within the hypomethylated regions showed allelic DNA methylation at one allele, and no methylation coupled with repressive chromatin marks H3K9me3 or H3K27me3 at the other allele [101]. The data from the experiment also suggest that global hypomethylation in breast cancer likely occurs through gradual loss of DNA methylation instead of an active mechanism involving demethylating enzymes, and that this gradual global hypomethylation most likely triggers the repressive chromatin domains [101].

Although hypomethylation of transcription regulatory regions in breast cancer is rare when compared to hypermethylation of CpG islands, it still does occur. For example, the gene encoding protease urokinase-type plasminogen activator (*PLAU/uPA*) is hypomethylated and over expressed in conjugation with tumour progression in breast cancers, with increased *uPA* expression resulting in increased tumour aggression and poor clinical outcome [102, 103]. A high-resolution analysis of DNA methylation in breast cancer also identified approximately 1500 regions that are hypomethylated [78, 104, 105]. Taken together, this evidence, in combination with the previous studies discussed, indicates that many of these hypomethylated regions may not only contribute to genomic instability, but probably contain genes, regulatory sequences, or mechanisms involved in breast cancer development.

### DNA methylation and circadian rhythms

Although not many studies have demonstrated a direct role for DNA methylation in the circadian clock, there is evidence suggesting that this epigenetic process may play a pivotal role in influencing circadian rhythms. Furthermore, through numerous studies, every core clock gene has been identified as being aberrantly methylated in various human malignancies [106]. Among these malignancies is breast cancer, with many studies illustrating methylation profiles of the core clock genes that are consistent with the aberrant expression patterns previously discussed.

A recent study investigated the role of DNA methylation on circadian behaviour by using a mouse model system and measuring the effect of altered day length on global DNA methylation within the SCN [107]. The results showed that mice entrained to a 22-hour day had an altered endogenous free-running period (FRP) and 1,294 differentially methylated regions when compared to mice entrained to a regular 24-hour day [107]. Among the differentially methylated regions were hypermethylated *Per2* and *Cry1* promoters, and hypomethylated *CLOCK* promoters [107]. To verify further the influence of DNA methylation on FRP, the experimenters inhibited methylation through the global DNMT inhibitor zebularine, and the results showed a significant suppression of FRP changes in the DNMT-inhibited mice. Finally, the study illustrated that the aberrant changes to FRP through DNA methylation are plastic, with short-day mice that are entrained back to a regular 24-hour day showing a reversion in FRP and DNA methylation levels [107]. These results represent the first evidence of a direct role for dynamic DNA methylation in the circadian clock, and illustrate that the activity of the enzymes catalyzing DNA methylation may be influenced by light-dependent induction or repression.

As discussed previously, the core clock genes can influence breast cancer development through a variety of mechanisms. It is therefore not surprising that promoter methylation profiles in breast cancer correlate with the oncogenic and oncostatic actions of the core clock genes. For example, a pair of studies showed that all the breast cancer types tested had *CLOCK*-promoter hypomethylation and *Cry2*-promoter hypermethylation when compared to controls [63, 72]. Another study illustrated that 95% of the breast cancer cells tested in women showed aberrant promoter methylation in the *Period* genes, including *Per1*- and *Per2*-promoter hypermethylation [108]. The methylation status of the *Per* genes was also linked to increased expression of *ERBB2*, the gene used to help classify breast cancers by type and a prognostic marker that negatively correlates with disease-free survival and overall survival [108, 109].

Even though there is some evidence for the influence of DNA methylation on circadian rhythms and a consistent promoter-methylation pattern between the core clock genes and breast cancer development, skepticism remains about the actual role of DNA methylation in circadian rhythms. The question remains whether aberrant DNA methylation at clock genes in cancer cells is the cause of circadian disruption, or simply a bystander effect of deregulated circadian rhythms. One theory, based on data from *Neurospora*, suggests that the aberrant methylation does not play a causative role and is instead a readout [106]. In either case, the epigenetic influence on CD-induced breast cancer likely extends into another epigenetic mechanism.

### MicroRNAs

Epigenetic control can also be mediated by means of small regulatory RNAs, specifically microRNAs (miRNAs). Mature miRNAs are abundant, small, single-stranded, noncoding RNAs that are potent regulators of gene expression [110]. Functional (mature) miRNAs can originate from either the exons or introns of non-coding genes, or introns of protein-coding genes [111–113]. The miRNAs found within the transcripts of other genes are under direct transcriptional control of those genes, while miRNAs found within intergenic regions are under their own control [111, 112]. Furthermore, miRNAs can be transcribed as a single unit or part of a polycistron, with polycistron miRNA expression acting as an efficient mechanism to target a single mRNA or to target multiple mRNAs in a signal molecular pathway [114–116].

The production and development of mature miRNAs in mammals involves multiple steps and numerous proteins and complexes [117]. First, transcription results in primary miRNA (pri-miRNA) in the nucleus, with the leading candidate for this pri-miRNA transcription being RNA polymerase (pol) II, but evidence showing that RNA pol III may also play a role in some transcripts [118, 119]. The RNase III endonuclease Drosha then forms a microprocessor complex with the protein DGCR8/Pasha, and digests the pri-miRNA to release a 60–70 nucleotide (nt) stem-loop intermediate known as precursor miRNA (pre-miRNA), with the base of the stem loop having a 5′ phosphate and a two nucleotide 3′ overhang that is characteristic of RNase III cleavage [120, 121]. The pre-miRNA is then transported to the cytoplasm by Ran-GTP and the export receptor Exportin5, where it undergoes more processing by the enzyme Dicer [121]. With the nuclear processing by Drosha defining one end of the pre-miRNA, the other end is processed by Dicer, and as this enzyme is also an RNase III endonuclease, the cleavage results in a 5′ phosphate and a two nucleotide 3′ overhang in place of the stem loop, as well as the formation of a mature double stranded miRNA duplex (miRNA:miRNA*) [121, 122].

In order to control the translation of their target mRNAs, the mature miRNA duplex associates with the RNA-induced silencing complex (RISC), which is composed of the proteins Argonaute (AGO), protein activator of the interferon-induced protein kinase (PACT), fragile X mental retardation protein (FXR), tudor staphylococcal nuclease (Tudor-SN), as well as other proteins [123]. However, evidence shows that the miRNA:miRNA* duplex is generally short-lived in comparison to the mature single-stranded miRNA, with fragments from the opposing arm (miRNA*) being found in much smaller quantities in libraries of cloned miRNAs [124, 125]. Although it appears that the AGO protein initiates the dissociation of one of the strands, the exact mechanism of strand selection in mammals has yet to be elucidated [116]. However, one theory suggests that the miRNA* is peeled away and degraded when the miRNA:miRNA* duplex is loaded into RISC because the other arm of the duplex has a less-tightly paired 5′ end, and as a result, it is more thermodynamically favourable to separate the duplex in this manner when association with RISC occurs [126].

Once the miRNA-RISC complex is assembled, it then binds to the 3′ UTR (untranslated region) of target mRNAs, resulting in posttranscriptional down regulation of gene expression by one of two mechanisms; mRNA cleavage or translational repression. If there is extensive complementarity between the miRNA and the 3′ UTR, then cleavage of the mRNA occurs [123]. However, if the homology between the miRNA and 3′ UTR is not sufficient, productive translation can be repressed through processing bodies (P-bodies) [127]. In mammals, binding is usually not very high in complementarity, so not only is suppression of gene expression by miRNAs usually achieved through inhibitory machinery, but each miRNA has numerous targets and can influence the expression of many different genes [128].

### MicroRNAs and breast cancer

The activity of mature miRNAs has an impact on a variety of cancer related processes, such as cellular differentiation, proliferation, apoptosis, and genome stability. One role by which miRNAs can influence these processes is by acting as oncogenic miRNAs (oncomiRs). Among the most well-studied and abundant oncomiRs in various cancers, including breast cancer, is miR-21 [116]. With its expression being linked to induction by signal transducer and activator of transcription 3 (STAT-3), a protein shown to be overexpressed in breast cancer and linked to various oncogenes, miR-21 is often overexpressed as well, resulting in the negative regulation of various tumour-suppressor genes [116, 129]. Specifically, miR-21 has been shown to down regulate the expression of the tumour suppressors phosphatase and tensin homolog (*PTEN*) and programmed cell death 4 (*PDCD_4_*) [130, 131]. In breast cancer, PTEN coordinates G_1_ cell-cycle arrest by down regulating the cyclin D1 protein and increasing p27 expression, causing reduced cell growth and increased apoptosis [132, 133]. PDCD_4_ inhibits CDK1 activity through induction of p21, and loss of PDCD_4_ is linked to breast cancer development [130, 134]. An experiment also showed that knockdown of miR-21 in MCF-7 and MDA-MB-231 breast cancer cell lines inhibited *in vitro* growth and migration, as well as *in vivo* growth [135].

The expression and activity of oncomiRs can also extend into miRNA clusters. For example, the miR-17–92 cluster was among the first miRNA groups discovered to be deregulated in various cancers, including breast cancer [116]. Containing six miRNAs that are transcribed together as a single polycistron, up-regulation of these miRNAs has been linked with increased levels of cell proliferation and lowered apoptosis [116]. Another oncomiR cluster, the miR-221/222 tandem, has been linked to ER-negative and basal-like breast cancers [136, 137]. In terms of ER-negative breast cancer, miR-221/222 was shown to target the 3′ UTR of ERα, resulting in lowered ERα protein levels in MCF-7 breast cancer cell lines and increased resistance to TAM treatment [137]. Basal-like breast cancer is notoriously more aggressive than all the other breast cancer types, and it seems that this aggressiveness may be linked to miR-221/222 expression [136]. Epithelial-to-mesenchymal transition (EMT) is the process by which epithelial cells lose the adherent properties and tight junctions that keep them in contact with neighbouring cells and gain mesenchymal properties, causing gene expression changes within the cells, resistance to apoptosis, and the ability to break through the basal membrane and migrate over long distances [138]. Evidence shows that the miR-221/222 cluster promotes EMT by repressing expression of the trichorhinophalangeal syndrome 1 (TRPS1) protein, which in turn results in an increase of the EMT-promoting protein zinc finger E-box binding homeobox 2 (ZEB2), thus potentially contributing to the more aggressive clinical behaviour of basal-like breast cancer [136].

In addition to acting as oncomiRs, miRNAs can also act as tumour suppressors. Several individual miRNAs have been identified as tumour suppressors in breast cancers. Among these are miR-99a and miR-140. MicroRNA 99a overexpression has been shown to induce G_1_ cell-cycle arrest and apoptosis by lowering expression of the mammalian target of rapamycin (*mTOR*) gene, with miR-99a being a potential candidate for use as a therapeutic strategy for effectively controlling breast cancer development [139]. The transcription factor sex determining region Y-box 2 (SOX2) has been reported to be overexpressed in breast cancers and identified as playing a role in the early steps of tumour initiation through cancer stem cells (CSCs) [140]. *SOX2* has been identified as a target of miR-140, and in ER-α breast cancer cells, estrogen stimulation lowers miR-140 expression through ER-α binding to an ERE flanking the mir-140 promoter, thus increasing levels of *SOX2* [141]. MicroRNA tumour-suppressor activity can also extend into miRNA clusters and result in more potent regulation than through individual miRNAs. For example, the miR-143/145 cluster has been shown to lower ERBB3 protein levels and cause less proliferation and invasion in breast cancer cells [142]. Interestingly, although the miRNAs are still capable of causing tumour suppression effects on their own, expression of the miR-143/145 cluster results in more potent tumour suppression through a synergistic effect [142].

### MicroRNAs and circadian rhythms

MicroRNA activity has been shown to be associated with circadian rhythms through rhythmically regulated miRNAs and through direct influence on the circadian cycle. Numerous studies have illustrated that miRNAs oscillate throughout a 24-hour circadian day. For example, in *Arabidopsis*, miRNAs-167, 168, 171, and 398 were shown to oscillate, based on environmental light; daytime levels were also reported as higher than nighttime levels, and the oscillation of these miRNAs was governed through photic control and not an internal clock [143]. A microarray-based study on mouse livers also revealed that over 13% of the probed miRNAs exhibited circadian expression patterns [144]. Among these miRNAs were miR-181d and 191, with an inversely-correlated expression pattern being observed between these miRNAs and their respective targets, *CLOCK* and *Bmal1* [144]. In another study conducted on HeLa cell lines, results showed that the miR-192/194 cluster directly regulates the entire *Period* gene family, with increased expression of the miR192/194 tandem causing lowered expression of the *Period* genes and a shortening of the circadian cycle [145].

Of the miRNAs involved in circadian rhythms, miR-132 may potentially play a very prominent role, especially when considering the link between circadian rhythms and breast cancer. A rodent model experiment showed that miR-132 exhibited oscillatory expression in wild-type mice, but not in circadian mutant mice [146]. Further investigation has also illustrated that miR-132 is induced by light within the SCN and that it has the capacity to entrain or reset the circadian clock [147]. The proposed model suggests that nocturnal light triggers the chromatin remodeling gene methyl CpG binding protein 2 (*MeCP2*), which triggers the transcription of various light-responsive genes, including the miR-132 promoter, *Per1*, *Per2*, PABP interacting protein 2A (*Paip2A*), and B-cell translocation family member 2 (*Btg2*) [147]. As PERIOD, PAIP2A, and BTG2 protein levels increase, PAIP2A and BTG2 levels eventually reach a certain level and *Per1* and *Per2* transcription is inhibited by these two proteins [147]. Simultaneously, levels of mature miR-132 also increase through miR-132 promoter transcription and processing of pri-miRNA into functional miR-132 [147]. As levels of mature miR-132 increase, translational repression of its targets *Mecp2*, *Paipa2A*, and *BTG2* eventually occurs, thus restoring homeostasis and resetting the induction caused to the circadian clock by nocturnal light [147].

Interestingly, a recent study has reported that miR-132 levels are lowered in breast cancer cells, with miR-132 being identified as a tumour suppressor by inhibiting proliferation, migration, invasion, and metastasis [148]. Therefore, by extension, the combination of light at night and increased breast cancer risk through shift work could potentially result in an aberrant compounded effect through lower miR-132 expression. Due to increased risk of breast cancer development, miR-132 levels may be lowered, and as a result, further nocturnal exposure to light would not only increase deregulation of the circadian clock due to the inhibition of homeostasis induced by miR-132, but also cause lowered oncostatic activity through increased transcriptional inhibition of the *Period* genes by PAIP2A and BTG2.

Although none of the research discussed thus far has investigated the link between epigenetics, circadian disruption, and breast cancer directly, the fact that the studies discussed have dealt with parts of this equation and illustrate potential overlap, demonstrates that a potentially substantial link may exist between all three factors.

## CD-INDUCED BREAST CANCER: AN EPIGENETIC LINK?

As discussed thus far, numerous studies have investigated the link between circadian rhythms and breast cancer, epigenetics and breast cancer, and epigenetics and circadian rhythms. However, to date, only a handful of studies have incorporated all three factors and investigated the link between CD-induced breast cancer and epigenetic modifications. One of these studies incorporated a rodent model to investigate global methylation, while the other studies were conducted on human blood samples and focused on the influence of shift work on DNA methylation in peripheral blood cells.

The rodent model study, conducted to investigate the link between CD-induced breast cancer and epigenetic modifications, involved measuring the effect of circadian disruption on global methylation levels and the effect of melatonin on this relationship [149]. Three groups were used in the study: a control group, a light at night (LAN) group, and a LAN plus MLT group, with all three of the groups being injected with the 4T1-murine breast cancer cell line. The results showed that the LAN group had ~30% less global methylation and larger tumour size when compared to the control group, while the LAN+MLT group only showed a ~10% decrease in global methylation and the smallest amount of tumour growth out of all three groups [149]. These results not only reiterated the important role that MLT plays in breast cancer development, but illustrated the important role that DNA methylation may play in CD-induced breast cancer and that melatonin levels may be linked to the aberrant DNA methylation that is occurring.

In a genome methylation analysis of 5,409 CpG sites on nurse blood samples, data showed that 3359 (66.4%) of the sites were hypermethylated and 1,816 (33.6%) were hypomethylated in long-term shift workers, as compared to daytime workers [150]. Many of these differentially methylated CpG sites were located near the promoter sequences of circadian-related and cancer-relevant genes. Specifically, aberrant methylation of the circadian genes *CLOCK* and *Cry2* was reported, with shift workers exhibiting *CLOCK* hypomethylation and *Cry2* hypermethylation when compared to day workers, a pattern that has been associated with breast cancer and discussed previously [63, 72]. In another study, a total of 50 CpG sites across 26 unique imprinted genes showed significant differences in methylation between shift workers and day workers, with a tendency towards more hypomethylation than hypermethylation [151]. The genes that showed the highest variation in methylation, distal-less homeobox 5 (*DLX5*) and tumour protein 73 (*TP73*), are genes that are both linked to cancer development [151]. The *DLX5* gene is a transcriptional factor that promotes cell proliferation through up-regulation of *c-MYC* promoter activity and its expression has been linked with breast cancer, while the *TP73* gene is an important component of the p53 family of cell-cycle regulatory proteins and is altered in the majority of cancers [152, 153]. Together, these two studies were among the first to illustrate that shift work can result in aberrant changes to DNA methylation at specific genes that are not only linked to circadian rhythms, but also breast cancer development.

The most recent human blood-analysis study conducted a genome-wide CpG island methylation assay of blood samples at miRNA promoters [154]. The results illustrated that 50 CpG loci, corresponding to 31 miRNAs, were differentially methylated in shift workers when compared to day workers. Among the differentially methylated genes was hypermethylation of the promoter for miR-219, a miRNA that has been linked to breast cancer development and has been identified as a potential regulator of circadian duration via the modulation of CLOCK- and BMAL1-dependent *Per* transcription [146, 154, 155]. To investigate further, the experimenters performed a genome-wide microarray on an overexpressed miR-219, MCF-7 breast cancer cell line, which identified 319 differentially expressed transcripts [154]. Among the cancer-relevant pathways affected by these changes were increased apoptosis and immunomediated anti-tumour activity. In a follow-up study, the experimenters investigated the role of another miRNA that showed significant promoter hypermethylation in the shift worker blood samples, miR-34b [156]. MCF-7 breast cancer cells were transfected with miR-34b, and 230 differentially expressed transcripts were identified, corresponding again to increased apoptosis and immunomediated anti-tumour activity. Even though these studies analyzed DNA methylation levels at specific locations like the previous experiments discussed, they were the first to illustrate that the epigenetic modifications involved in CD-induced breast cancer may extend into miRNA regulation.

## FUTURE DIRECTIONS AND CONCLUSIONS

Although these handful of studies have provided valuable insights into the role of epigenetics in CD-induced breast cancer, there are glaring omissions and new directions that need to be investigated. First, none of the studies investigated the effect of CD on breast cancer development in mammary tissues. Second, although the experiment performed by Schwimmer et al. [149] did incorporate a circadian disruption scheme on a rodent model, the CD scheme was not very in-depth because it did not incorporate varying degrees of CD, and the effect of CD was skewed due to artificial MLT levels. Third, all the studies focused on DNA methylation profiles, and although the Shi et al. [154] study identified aberrant DNA methylation at miRNA promoters, it did not measure direct changes in small RNA expression due to CD. Fourth, none of the studies considered the potential fluctuations in epigenetic modifications due to varying time points within a circadian cycle. Finally, although the studies illustrated epigenetic changes due to CD and their potential role in breast cancer development, none of the studies showed direct downstream consequences due to these epigenetic modifications. Therefore, to shed new light on the epigenetic modifications involved in CD-induced breast cancer, focus needs to be placed on investigating direct CD-induced changes in DNA methylation and miRNA levels in mammary tissues. To accomplish this, environmentally controlled experiments utilizing model systems and incorporating varying degrees of circadian disruption, mammary tissue extractions at various time points following CD, and mammary tissue extractions at specific time points within the circadian cycle, need to be performed. Although incorporating a murine breast cancer model has its drawbacks, such as the difficulties associated with interpreting the data because of the variation amongst the different models, or the fact that no single mouse model shows all the expression patterns and characteristics of human breast cancer, incorporating in-depth CD schemes on rodent model systems is the next logical extension [157]. This approach will allow for a closer investigation into the dynamic and influence of CD on breast cancer development, and new insights and directions may be generated that will lead to a deeper understanding of the potential carcinogenicity of varying degrees of CD, influence of epigenetic circadian fluctuations on tumourigenesis, and the direct effect of CD on epigenetic processes in mammary tissues.

In summary, several conclusions can be drawn from the existing literature:

- Circadian rhythms play a role in breast cancer development. Aberrant exposure to light during the dark phase of the circadian cycle can disrupt the activity of the SCN and cause aberrant changes to downstream processes that influence breast cancer. Among these processes are nocturnal MLT and core clock gene activity, with lower nocturnal MLT levels causing oncogenic effects and the core clock genes influencing a variety of cellular processes related to breast cancer.
- The role of epigenetics has been recognized increasingly in the etiology of breast cancer. Two epigenetic mechanisms, DNA methylation and miRNA activity, influence a variety of cellular processes related to breast cancer development. In addition, these two epigenetic processes have also been linked to circadian rhythms. DNA methylation has been shown to regulate core clock gene expression and may also potentially play a direct role in the circadian clock. While miRNAs have been shown to oscillate with the circadian cycle and directly influence components of the circadian clock.
- Although literature has provided evidence for a link between epigenetic modifications and circadian rhythms, with a clear overlap existing between these two processes and breast cancer development, only a handful of studies have incorporated all three factors and directly studied the epigenetic modifications involved in CD-induced breast cancer. Although these studies represent a good start, there are still many aspects and different directions that need to be addressed when investigating the epigenetic modifications involved in CD-induced breast cancer.

## References

[1] IARC (2012). World cancer factsheet.

[2] AmericanCancerSociety (2015). Breast Cancer in Men. Cancer Facts and Figures 2015.

[3] Giordano SH, Cohen DS, Buzdar AU, Perkins G, Hortobagyi GN (2004). Breast carcinoma in men: a population-based study. Cancer.

[4] Abeloff MD, Armitage JO, Niederhuber JE, Kastan MB, McKenna WG (2008). Abeloff's Clinical Oncology.

[5] Shackney SE, Silverman JF (2003). Molecular evolutionary patterns in breast cancer. Advances in anatomic pathology.

[6] Simpson PT, Reis-Filho JS, Gale T, Lakhani SR (2005). Molecular evolution of breast cancer. The Journal of pathology.

[7] Britten A, Rossier C, Taright N, Ezra P, Bourgier C (2013). Genomic classifications and radiotherapy for breast cancer. European journal of pharmacology.

[8] Li Y, Meeran SM, Patel SN, Chen H, Hardy TM, Tollefsbol TO (2013). Epigenetic reactivation of estrogen receptor-alpha (ERalpha) by genistein enhances hormonal therapy sensitivity in ERalpha-negative breast cancer. Molecular cancer.

[9] Mourits MJ, De Vries EG, Willemse PH, Ten Hoor KA, Hollema H, Van der Zee AG (2001). Tamoxifen treatment and gynecologic side effects: a review. Obstetrics and gynecology.

[10] Gadducci A, Biglia N, Sismondi P, Genazzani AR (2005). Breast cancer and sex steroids: critical review of epidemiological, experimental and clinical investigations on etiopathogenesis, chemoprevention and endocrine treatment of breast cancer. Gynecological endocrinology: the official journal of the International Society of Gynecological Endocrinology.

[11] Jang ER, Lim SJ, Lee ES, Jeong G, Kim TY, Bang YJ, Lee JS (2004). The histone deacetylase inhibitor trichostatin A sensitizes estrogen receptor alpha-negative breast cancer cells to tamoxifen. Oncogene.

[12] Yang X, Phillips DL, Ferguson AT, Nelson WG, Herman JG, Davidson NE (2001). Synergistic activation of functional (ER)-alpha by DNA methyltransferase and histone deacetylase inhibition in human ER-alpha-negative breast cancer cells. Cancer research.

[13] Hansen J, Stevens RG (2012). Case-control study of shift-work and breast cancer risk in Danish nurses: impact of shift systems. European journal of cancer.

[14] Cajochen C, Frey S, Anders D, Spati J, Bues M, Pross A, Mager R, Wirz-Justice A, Stefani O (2011). Evening exposure to a light-emitting diodes (LED)-backlit computer screen affects circadian physiology and cognitive performance. Journal of applied physiology (Bethesda, Md : 1985).

[15] Filipski E, Delaunay F, King VM, Wu MW, Claustrat B, Grechez-Cassiau A, Guettier C, Hastings MH, Francis L (2004). Effects of chronic jet lag on tumor progression in mice. Cancer research.

[16] Gooley JJ, Chamberlain K, Smith KA, Khalsa SB, Rajaratnam SM, Van Reen E, Zeitzer JM, Czeisler CA, Lockley SW (2011). Exposure to room light before bedtime suppresses melatonin onset and shortens melatonin duration in humans. The Journal of clinical endocrinology and metabolism.

[17] Mallis MM, DeRoshia CW (2005). Circadian rhythms, sleep, and performance in space. Aviation, space, and environmental medicine.

[18] Wirz-Justice A, Bromundt V, Cajochen C Circadian Disruption and Psychiatric Disorders: The Importance of Entrainment. Sleep Medicine Clinics.

[19] Flynn-Evans EE, Mucci L, Stevens RG, Lockley SW (2013). Shiftwork and prostate-specific antigen in the National Health and Nutrition Examination Survey. Journal of the National Cancer Institute.

[20] Sigurdardottir LG, Valdimarsdottir UA, Mucci LA, Fall K, Rider JR, Schernhammer E, Czeisler CA, Launer L, Harris T, Stampfer MJ, Gudnason V, Lockley SW (2013). Sleep disruption among older men and risk of prostate cancer. Cancer epidemiology, biomarkers & prevention: a publication of the American Association for Cancer Research, cosponsored by the American Society of Preventive Oncology.

[21] Vinogradova IA, Anisimov VN, Bukalev AV, Semenchenko AV, Zabezhinski MA (2009). Circadian disruption induced by light-at-night accelerates aging and promotes tumorigenesis in rats. Aging.

[22] Knutsson A, Alfredsson L, Karlsson B, Akerstedt T, Fransson EI, Westerholm P, Westerlund H (2013). Breast cancer among shift workers: results of the WOLF longitudinal cohort study. Scandinavian journal of work, environment & health.

[23] Demers PA, Wong I, McLeod C The prevelance of shift work in Canada.

[24] Rabstein S, Harth V, Pesch B, Pallapies D, Lotz A, Justenhoven C, Baisch C, Schiffermann M, Haas S, Fischer HP, Heinze E, Pierl C, Brauch H, Hamann U, Ko Y, Bruning T (2013). Night work and breast cancer estrogen receptor status—results from the German GENICA study. Scandinavian journal of work, environment & health.

[25] Moore RY (1983). Organization and function of a central nervous system circadian oscillator: the suprachiasmatic hypothalamic nucleus. Federation proceedings.

[26] Zelinski EL, Deibel SH, McDonald RJ (2014). The trouble with circadian clock dysfunction: multiple deleterious effects on the brain and body. Neuroscience and biobehavioral reviews.

[27] Haus EL, Smolensky MH (2013). Shift work and cancer risk: potential mechanistic roles of circadian disruption, light at night, and sleep deprivation. Sleep medicine reviews.

[28] Reppert SM, Weaver DR (2002). Coordination of circadian timing in mammals. Nature.

[29] Antle MC, Silver R (2005). Orchestrating time: arrangements of the brain circadian clock. Trends in neurosciences.

[30] Abrahamson EE, Moore RY (2001). Suprachiasmatic nucleus in the mouse: retinal innervation, intrinsic organization and efferent projections. Brain research.

[31] Welsh DK, Takahashi JS, Kay SA (2010). Suprachiasmatic nucleus: cell autonomy and network properties. Annual review of physiology.

[32] Butler MP, Silver R (2009). Basis of robustness and resilience in the suprachiasmatic nucleus: individual neurons form nodes in circuits that cycle daily. Journal of biological rhythms.

[33] Silver R, Romero MT, Besmer HR, Leak R, Nunez JM, LeSauter J (1996). Calbindin-D28K cells in the hamster SCN express light-induced Fos. Neuroreport.

[34] Monsees GM, Kraft P, Hankinson SE, Hunter DJ, Schernhammer ES (2012). Circadian genes and breast cancer susceptibility in rotating shift workers. International journal of cancer Journal international du cancer.

[35] Folkard S (2008). Do permanent night workers show circadian adjustment? A review based on the endogenous melatonin rhythm. Chronobiology international.

[36] Haus E, Smolensky M (2006). Biological clocks and shift work: circadian dysregulation and potential long-term effects. Cancer causes & control: CCC.

[37] Korkmaz A, Topal T, Tan DX, Reiter RJ (2009). Role of melatonin in metabolic regulation. Reviews in endocrine & metabolic disorders.

[38] Arendt J (2006). Melatonin and human rhythms. Chronobiology international.

[39] Blask DE (2009). Melatonin, sleep disturbance and cancer risk. Sleep medicine reviews.

[40] Dauchy RT, Xiang S, Mao L, Brimer S, Wren MA, Yuan L, Anbalagan M, Hauch A, Frasch T, Rowan BG, Blask DE, Hill SM (2014). Circadian and melatonin disruption by exposure to light at night drives intrinsic resistance to tamoxifen therapy in breast cancer. Cancer research.

[41] Kothari A, Borges A, Ingle A, Kothari L (1997). Combination of melatonin and tamoxifen as a chemoprophylaxis against N-nitroso-N-methylurea-induced rat mammary tumors. Cancer letters.

[42] Wilson ST, Blask DE, Lemus-Wilson AM (1992). Melatonin augments the sensitivity of MCF-7 human breast cancer cells to tamoxifen *in vitro*. The Journal of clinical endocrinology and metabolism.

[43] Levi F (2006). Chronotherapeutics: the relevance of timing in cancer therapy. Cancer causes & control: CCC.

[44] Blumenthal RD, Waskewich C, Goldenberg DM, Lew W, Flefleh C, Burton J (2001). Chronotherapy and chronotoxicity of the cyclooxygenase-2 inhibitor, celecoxib, in athymic mice bearing human breast cancer xenografts. Clinical cancer research: an official journal of the American Association for Cancer Research.

[45] Depres-Brummer P, Berthault-Cvitkovic F, Levi F, Brienza S, Vannetzel JM, Jasmin C, Misset JL (1995). Circadian rhythm-modulated (CRM) chemotherapy of metastatic breast cancer with mitoxantrone, 5-fluorouracil, and folinic acid: preliminary results of a phase I trial. The Journal of infusional chemotherapy.

[46] Reiter RJ (2004). Mechanisms of cancer inhibition by melatonin. Journal of pineal research.

[47] Blask DE, Dauchy RT, Sauer LA, Krause JA, Brainard GC (2003). Growth and fatty acid metabolism of human breast cancer (MCF-7) xenografts in nude rats: impact of constant light-induced nocturnal melatonin suppression. Breast cancer research and treatment.

[48] Cos S, Mediavilla D, Martinez-Campa C, Gonzalez A, Alonso-Gonzalez C, Sanchez-Barcelo EJ (2006). Exposure to light-at-night increases the growth of DMBA-induced mammary adenocarcinomas in rats. Cancer letters.

[49] Sauer LA, Dauchy RT, Blask DE (2001). Melatonin inhibits fatty acid transport in inguinal fat pads of hepatoma 7288CTC-bearing and normal Buffalo rats via receptor-mediated signal transduction. Life sciences.

[50] Benitez-King G, Huerto-Delgadillo L, Anton-Tay F (1993). Binding of 3H-melatonin to calmodulin. Life sciences.

[51] Coticchia CM, Revankar CM, Deb TB, Dickson RB, Johnson MD (2009). Calmodulin modulates Akt activity in human breast cancer cell lines. Breast cancer research and treatment.

[52] Soto-Vega E, Meza I, Ramirez-Rodriguez G, Benitez-King G (2004). Melatonin stimulates calmodulin phosphorylation by protein kinase C. Journal of pineal research.

[53] Storch KF, Lipan O, Leykin I, Viswanathan N, Davis FC, Wong WH, Weitz CJ (2002). Extensive and divergent circadian gene expression in liver and heart. Nature.

[54] Albrecht U, Ripperger JA Clock Genes. Encyclopedia of Neuroscience: Springer Berlin Heidelberg.

[55] Ko CH, Takahashi JS (2006). Molecular components of the mammalian circadian clock. Human molecular genetics.

[56] Eide EJ, Woolf MF, Kang H, Woolf P, Hurst W, Camacho F, Vielhaber EL, Giovanni A, Virshup DM (2005). Control of mammalian circadian rhythm by CKIepsilon-regulated proteasome-mediated PER2 degradation. Molecular and cellular biology.

[57] Lowrey PL, Shimomura K, Antoch MP, Yamazaki S, Zemenides PD, Ralph MR, Menaker M, Takahashi JS (2000). Positional syntenic cloning and functional characterization of the mammalian circadian mutation tau. Science.

[58] Nakamura W, Yamazaki S, Takasu NN, Mishima K, Block GD (2005). Differential response of Period 1 expression within the suprachiasmatic nucleus. The Journal of neuroscience: the official journal of the Society for Neuroscience.

[59] Gery S, Komatsu N, Baldjyan L, Yu A, Koo D, Koeffler HP (2006). The circadian gene per1 plays an important role in cell growth and DNA damage control in human cancer cells. Molecular cell.

[60] Gery S, Virk RK, Chumakov K, Yu A, Koeffler HP (2007). The clock gene Per2 links the circadian system to the estrogen receptor. Oncogene.

[61] Parl FF, Dawling S, Roodi N, Crooke PS (2009). Estrogen metabolism and breast cancer: a risk model. Annals of the New York Academy of Sciences.

[62] Xiao L, Chang AK, Zang MX, Bi H, Li S, Wang M, Xing X, Wu H (2014). Induction of the CLOCK gene by E2-ERalpha signaling promotes the proliferation of breast cancer cells. PloS one.

[63] Hoffman AE, Yi CH, Zheng T, Stevens RG, Leaderer D, Zhang Y, Holford TR, Hansen J, Paulson J, Zhu Y (2010). CLOCK in breast tumorigenesis: genetic, epigenetic, and transcriptional profiling analyses. Cancer research.

[64] Jung CH, Kim EM, Park JK, Hwang SG, Moon SK, Kim WJ, Um HD (2013). Bmal1 suppresses cancer cell invasion by blocking the phosphoinositide 3-kinase-Akt-MMP-2 signaling pathway. Oncology reports.

[65] Zeng ZL, Wu MW, Sun J, Sun YL, Cai YC, Huang YJ, Xian LJ (2010). Effects of the biological clock gene Bmal1 on tumour growth and anti-cancer drug activity. Journal of biochemistry.

[66] Wang C, Fan S, Li Z, Fu M, Rao M, Ma Y, Lisanti MP, Albanese C, Katzenellenbogen BS, Kushner PJ, Weber B, Rosen EM, Pestell RG (2005). Cyclin D1 antagonizes BRCA1 repression of estrogen receptor alpha activity. Cancer research.

[67] Fu L, Pelicano H, Liu J, Huang P, Lee C (2002). The circadian gene Period2 plays an important role in tumor suppression and DNA damage response *in vivo*. Cell.

[68] Chen-Goodspeed M, Lee CC (2007). Tumor suppression and circadian function. Journal of biological rhythms.

[69] Matsuo T, Yamaguchi S, Mitsui S, Emi A, Shimoda F, Okamura H (2003). Control mechanism of the circadian clock for timing of cell division *in vivo*. Science.

[70] Vriend LE, De Witt Hamer PC, Van Noorden CJ, Wurdinger T (2013). WEE1 inhibition and genomic instability in cancer. Biochimica et biophysica acta.

[71] Parker LL, Piwnica-Worms H (1992). Inactivation of the p34cdc2-cyclin B complex by the human WEE1 tyrosine kinase. Science.

[72] Hoffman AE, Zheng T, Yi CH, Stevens RG, Ba Y, Zhang Y, Leaderer D, Holford T, Hansen J, Zhu Y (2010). The core circadian gene Cryptochrome 2 influences breast cancer risk, possibly by mediating hormone signaling. Cancer prevention research.

[73] Klose RJ, Bird AP (2006). Genomic DNA methylation: the mark and its mediators. Trends in biochemical sciences.

[74] Liang G, Chan MF, Tomigahara Y, Tsai YC, Gonzales FA, Li E, Laird PW, Jones PA (2002). Cooperativity between DNA methyltransferases in the maintenance methylation of repetitive elements. Molecular and cellular biology.

[75] Goll MG, Bestor TH (2005). Eukaryotic cytosine methyltransferases. Annual review of biochemistry.

[76] Hata K, Okano M, Lei H, Li E (2002). Dnmt3L cooperates with the Dnmt3 family of de novo DNA methyltransferases to establish maternal imprints in mice. Development (Cambridge, England).

[77] Weber M, Schubeler D (2007). Genomic patterns of DNA methylation: targets and function of an epigenetic mark. Current opinion in cell biology.

[78] Jovanovic J, Ronneberg JA, Tost J, Kristensen V (2010). The epigenetics of breast cancer. Molecular oncology.

[79] De Smet C, Loriot A, Boon T (2004). Promoter-dependent mechanism leading to selective hypomethylation within the 5′ region of gene MAGE-A1 in tumor cells. Molecular and cellular biology.

[80] Merlo A, Herman JG, Mao L, Lee DJ, Gabrielson E, Burger PC, Baylin SB, Sidransky D (1995). 5′ CpG island methylation is associated with transcriptional silencing of the tumour suppressor p16/CDKN2/MTS1 in human cancers. Nature medicine.

[81] Jiang G, Plo I, Wang T, Rahman M, Cho JH, Yang E, Lopez BS, Xia F (2013). BRCA1-Ku80 protein interaction enhances end-joining fidelity of chromosomal double-strand breaks in the G1 phase of the cell cycle. The Journal of biological chemistry.

[82] Lieberman HB, Bernstock JD, Broustas CG, Hopkins KM, Leloup C, Zhu A (2011). The role of RAD9 in tumorigenesis. Journal of molecular cell biology.

[83] Veeck J, Wild PJ, Fuchs T, Schuffler PJ, Hartmann A, Knuchel R, Dahl E (2009). Prognostic relevance of Wnt-inhibitory factor-1 (WIF1) and Dickkopf-3 (DKK3) promoter methylation in human breast cancer. BMC cancer.

[84] Lamb R, Ablett MP, Spence K, Landberg G, Sims AH, Clarke RB (2013). Wnt pathway activity in breast cancer sub-types and stem-like cells. PloS one.

[85] Chen H, Chung S, Sukumar S (2004). HOXA5-induced apoptosis in breast cancer cells is mediated by caspases 2 and 8. Molecular and cellular biology.

[86] Salminen A, Kauppinen A, Hiltunen M, Kaarniranta K (2014). Epigenetic regulation of ASC/TMS1 expression: potential role in apoptosis and inflammasome function. Cellular and molecular life sciences: CMLS.

[87] Euhus DM, Bu D, Milchgrub S, Xie XJ, Bian A, Leitch AM, Lewis CM (2008). DNA methylation in benign breast epithelium in relation to age and breast cancer risk. Cancer epidemiology, biomarkers & prevention: a publication of the American Association for Cancer Research, cosponsored by the American Society of Preventive Oncology.

[88] Ferguson AT, Lapidus RG, Baylin SB, Davidson NE (1995). Demethylation of the estrogen receptor gene in estrogen receptor-negative breast cancer cells can reactivate estrogen receptor gene expression. Cancer research.

[89] Lapidus RG, Ferguson AT, Ottaviano YL, Parl FF, Smith HS, Weitzman SA, Baylin SB, Issa JP, Davidson NE (1996). Methylation of estrogen and progesterone receptor gene 5′ CpG islands correlates with lack of estrogen and progesterone receptor gene expression in breast tumors. Clinical cancer research: an official journal of the American Association for Cancer Research.

[90] Weigel RJ, deConinck EC (1993). Transcriptional control of estrogen receptor in estrogen receptor-negative breast carcinoma. Cancer research.

[91] Izadi P, Noruzinia M, Karimipoor M, Karbassian MH, Akbari MT (2012). Promoter hypermethylation of estrogen receptor alpha gene is correlated to estrogen receptor negativity in Iranian patients with sporadic breast cancer. Cell journal.

[92] Feng W, Shen L, Wen S, Rosen DG, Jelinek J, Hu X, Huan S, Huang M, Liu J, Sahin AA, Hunt KK, Bast RC, Shen Y, Issa JP, Yu Y (2007). Correlation between CpG methylation profiles and hormone receptor status in breast cancers. Breast cancer research: BCR.

[93] Ehrlich M (2009). DNA hypomethylation in cancer cells. Epigenomics.

[94] Feinberg AP, Vogelstein B (1983). Hypomethylation distinguishes genes of some human cancers from their normal counterparts. Nature.

[95] Brena RM, Costello JF (2007). Genome-epigenome interactions in cancer. Human molecular genetics.

[96] Hormozdiari F, Alkan C, Ventura M, Hajirasouliha I, Malig M, Hach F, Yorukoglu D, Dao P, Bakhshi M, Sahinalp SC, Eichler EE (2011). Alu repeat discovery and characterization within human genomes. Genome research.

[97] Lander ES, Linton LM, Birren B, Nusbaum C, Zody MC, Baldwin J, Devon K, Dewar K, Doyle M, FitzHugh W, Funke R, Gage D, Harris K, Heaford A, Howland J, Kann L (2001). Initial sequencing and analysis of the human genome. Nature.

[98] Park SY, Seo AN, Jung HY, Gwak JM, Jung N, Cho NY, Kang GH (2014). Alu and LINE-1 hypomethylation is associated with HER2 enriched subtype of breast cancer. PloS one.

[99] Kitkumthorn N, Mutirangura A (2011). Long interspersed nuclear element-1 hypomethylation in cancer: biology and clinical applications. Clinical epigenetics.

[100] Lister R, Pelizzola M, Dowen RH, Hawkins RD, Hon G, Tonti-Filippini J, Nery JR, Lee L, Ye Z, Ngo QM, Edsall L, Antosiewicz-Bourget J, Stewart R, Ruotti V, Millar AH, Thomson JA (2009). Human DNA methylomes at base resolution show widespread epigenomic differences. Nature.

[101] Hon GC, Hawkins RD, Caballero OL, Lo C, Lister R, Pelizzola M, Valsesia A, Ye Z, Kuan S, Edsall LE, Camargo AA, Stevenson BJ, Ecker JR, Bafna V, Strausberg RL, Simpson AJ (2012). Global DNA hypomethylation coupled to repressive chromatin domain formation and gene silencing in breast cancer. Genome research.

[102] Annecke K, Schmitt M, Euler U, Zerm M, Paepke D, Paepke S, von Minckwitz G, Thomssen C, Harbeck N (2008). uPA and PAI-1 in breast cancer: review of their clinical utility and current validation in the prospective NNBC-3 trial. Advances in clinical chemistry.

[103] Pakneshan P, Tetu B, Rabbani SA (2004). Demethylation of urokinase promoter as a prognostic marker in patients with breast carcinoma. Clinical cancer research: an official journal of the American Association for Cancer Research.

[104] Novak P, Jensen T, Oshiro MM, Watts GS, Kim CJ, Futscher BW (2008). Agglomerative epigenetic aberrations are a common event in human breast cancer. Cancer research.

[105] Shann YJ, Cheng C, Chiao CH, Chen DT, Li PH, Hsu MT (2008). Genome-wide mapping and characterization of hypomethylated sites in human tissues and breast cancer cell lines. Genome research.

[106] Joska TM, Zaman R, Belden WJ (2014). Regulated DNA methylation and the circadian clock: implications in cancer. Biology.

[107] Azzi A, Dallmann R, Casserly A, Rehrauer H, Patrignani A, Maier B, Kramer A, Brown SA (2014). Circadian behavior is light-reprogrammed by plastic DNA methylation. Nature neuroscience.

[108] Chen ST, Choo KB, Hou MF, Yeh KT, Kuo SJ, Chang JG (2005). Deregulated expression of the PER1, PER2 and PER3 genes in breast cancers. Carcinogenesis.

[109] Tsutsui S, Ohno S, Murakami S, Hachitanda Y, Oda S (2002). Prognostic value of c-erbB2 expression in breast cancer. Journal of surgical oncology.

[110] Hwang HW, Mendell JT (2006). MicroRNAs in cell proliferation, cell death, and tumorigenesis. British journal of cancer.

[111] Liu CG, Calin GA, Volinia S, Croce CM (2008). MicroRNA expression profiling using microarrays. Nature protocols.

[112] Liu CG, Spizzo R, Calin GA, Croce CM (2008). Expression profiling of microRNA using oligo DNA arrays. Methods (San Diego, Calif).

[113] Rodriguez A, Griffiths-Jones S, Ashurst JL, Bradley A (2004). Identification of mammalian microRNA host genes and transcription units. Genome research.

[114] Fujita S, Iba H (2008). Putative promoter regions of miRNA genes involved in evolutionarily conserved regulatory systems among vertebrates. Bioinformatics (Oxford, England).

[115] Griffiths-Jones S, Saini HK, van Dongen S, Enright AJ (2008). miRBase: tools for microRNA genomics. Nucleic acids research.

[116] Koturbash I, Zemp FJ, Pogribny I, Kovalchuk O (2011). Small molecules with big effects: the role of the microRNAome in cancer and carcinogenesis. Mutation research.

[117] Ranganna K, Mathew OP, Milton SG, Hayes BE (2013). MicroRNAome of Vascular Smooth Muscle Cells: Potential for MicroRNA-Based Vascular Therapies.

[118] Chen CZ, Li L, Lodish HF, Bartel DP (2004). MicroRNAs modulate hematopoietic lineage differentiation. Science.

[119] Lee Y, Kim M, Han J, Yeom KH, Lee S, Baek SH, Kim VN (2004). MicroRNA genes are transcribed by RNA polymerase II. The EMBO journal.

[120] Krol J, Loedige I, Filipowicz W (2010). The widespread regulation of microRNA biogenesis, function and decay. Nature reviews Genetics.

[121] Lee Y, Jeon K, Lee JT, Kim S, Kim VN (2002). MicroRNA maturation: stepwise processing and subcellular localization. The EMBO journal.

[122] Lee Y, Ahn C, Han J, Choi H, Kim J, Yim J, Lee J, Provost P, Radmark O, Kim S, Kim VN (2003). The nuclear RNase III Drosha initiates microRNA processing. Nature.

[123] Hutvagner G, Zamore PD (2002). RNAi: nature abhors a double-strand. Current opinion in genetics & development.

[124] Lagos-Quintana M, Rauhut R, Yalcin A, Meyer J, Lendeckel W, Tuschl T (2002). Identification of tissue-specific microRNAs from mouse. Current biology: CB.

[125] Lim LP, Lau NC, Weinstein EG, Abdelhakim A, Yekta S, Rhoades MW, Burge CB, Bartel DP (2003). The microRNAs of Caenorhabditis elegans. Genes & development.

[126] Khvorova A, Reynolds A, Jayasena SD (2003). Functional siRNAs and miRNAs exhibit strand bias. Cell.

[127] Liu J, Valencia-Sanchez MA, Hannon GJ, Parker R (2005). MicroRNA-dependent localization of targeted mRNAs to mammalian P-bodies. Nature cell biology.

[128] Thomson DW, Bracken CP, Goodall GJ (2011). Experimental strategies for microRNA target identification. Nucleic acids research.

[129] Chen Y, Wang J, Wang X, Liu X, Li H, Lv Q, Zhu J, Wei B, Tang Y (2013). STAT3, a Poor Survival Predicator, Is Associated with Lymph Node Metastasis from Breast Cancer. Journal of breast cancer.

[130] Frankel LB, Christoffersen NR, Jacobsen A, Lindow M, Krogh A, Lund AH (2008). Programmed cell death 4 (PDCD4) is an important functional target of the microRNA miR-21 in breast cancer cells. The Journal of biological chemistry.

[131] Gong C, Yao Y, Wang Y, Liu B, Wu W, Chen J, Su F, Yao H, Song E (2011). Up-regulation of miR-21 mediates resistance to trastuzumab therapy for breast cancer. The Journal of biological chemistry.

[132] Lu Y, Lin YZ, LaPushin R, Cuevas B, Fang X, Yu SX, Davies MA, Khan H, Furui T, Mao M, Zinner R, Hung MC, Steck P, Siminovitch K, Mills GB (1999). The PTEN/MMAC1/TEP tumor suppressor gene decreases cell growth and induces apoptosis and anoikis in breast cancer cells. Oncogene.

[133] Weng LP, Brown JL, Eng C (2001). PTEN coordinates G(1) arrest by down-regulating cyclin D1 via its protein phosphatase activity and up-regulating p27 via its lipid phosphatase activity in a breast cancer model. Human molecular genetics.

[134] Goke R, Barth P, Schmidt A, Samans B, Lankat-Buttgereit B (2004). Programmed cell death protein 4 suppresses CDK1/cdc2 via induction of p21(Waf1/Cip1). American journal of physiology Cell physiology.

[135] Yan LX, Wu QN, Zhang Y, Li YY, Liao DZ, Hou JH, Fu J, Zeng MS, Yun JP, Wu QL, Zeng YX, Shao JY (2011). Knockdown of miR-21 in human breast cancer cell lines inhibits proliferation, *in vitro* migration and *in vivo* tumor growth. Breast cancer research: BCR.

[136] Stinson S, Lackner MR, Adai AT, Yu N, Kim HJ, O'Brien C, Spoerke J, Jhunjhunwala S, Boyd Z, Januario T, Newman RJ, Yue P, Bourgon R, Modrusan Z, Stern HM, Warming S (2011). TRPS1 targeting by miR-221/222 promotes the epithelial-to-mesenchymal transition in breast cancer. Science signaling.

[137] Zhao JJ, Lin J, Yang H, Kong W, He L, Ma X, Coppola D, Cheng JQ (2008). MicroRNA-221/222 negatively regulates estrogen receptor alpha and is associated with tamoxifen resistance in breast cancer. The Journal of biological chemistry.

[138] Kalluri R, Weinberg RA (2009). The basics of epithelial-mesenchymal transition. The Journal of clinical investigation.

[139] Hu Y, Zhu Q, Tang L (2014). MiR-99a antitumor activity in human breast cancer cells through targeting of mTOR expression. PloS one.

[140] Leis O, Eguiara A, Lopez-Arribillaga E, Alberdi MJ, Hernandez-Garcia S, Elorriaga K, Pandiella A, Rezola R, Martin AG (2012). Sox2 expression in breast tumours and activation in breast cancer stem cells. Oncogene.

[141] Zhang Y, Eades G, Yao Y, Li Q, Zhou Q (2012). Estrogen receptor alpha signaling regulates breast tumor-initiating cells by down-regulating miR-140 which targets the transcription factor SOX2. The Journal of biological chemistry.

[142] Yan X, Chen X, Liang H, Deng T, Chen W, Zhang S, Liu M, Gao X, Liu Y, Zhao C, Wang X, Wang N, Li J, Liu R, Zen K, Zhang CY (2014). miR-143 and miR-145 synergistically regulate ERBB3 to suppress cell proliferation and invasion in breast cancer. Molecular cancer.

[143] Sire C, Moreno AB, Garcia-Chapa M, Lopez-Moya JJ, San Segundo B (2009). Diurnal oscillation in the accumulation of Arabidopsis microRNAs, miR167, miR168, miR171 and miR398. FEBS letters.

[144] Na YJ, Sung JH, Lee SC, Lee YJ, Choi YJ, Park WY, Shin HS, Kim JH (2009). Comprehensive analysis of microRNA-mRNA co-expression in circadian rhythm. Experimental & molecular medicine.

[145] Nagel R, Clijsters L, Agami R (2009). The miRNA-192/194 cluster regulates the Period gene family and the circadian clock. The FEBS journal.

[146] Cheng HY, Papp JW, Varlamova O, Dziema H, Russell B, Curfman JP, Nakazawa T, Shimizu K, Okamura H, Impey S, Obrietan K (2007). microRNA modulation of circadian-clock period and entrainment. Neuron.

[147] Alvarez-Saavedra M, Antoun G, Yanagiya A, Oliva-Hernandez R, Cornejo-Palma D, Perez-Iratxeta C, Sonenberg N, Cheng HY (2011). miRNA-132 orchestrates chromatin remodeling and translational control of the circadian clock. Human molecular genetics.

[148] Zhang ZG, Chen WX, Wu YH, Liang HF, Zhang BX (2014). MiR-132 prohibits proliferation, invasion, migration, and metastasis in breast cancer by targeting HN1. Biochemical and biophysical research communications.

[149] Schwimmer H, Metzer A, Pilosof Y, Szyf M, Machnes ZM, Fares F, Harel O, Haim A (2014). Light at night and melatonin have opposite effects on breast cancer tumors in mice assessed by growth rates and global DNA methylation. Chronobiology international.

[150] Zhu Y, Stevens RG, Hoffman AE, Tjonneland A, Vogel UB, Zheng T, Hansen J (2011). Epigenetic impact of long-term shiftwork: pilot evidence from circadian genes and whole-genome methylation analysis. Chronobiology international.

[151] Jacobs DI, Hansen J, Fu A, Stevens RG, Tjonneland A, Vogel UB, Zheng T, Zhu Y (2013). Methylation alterations at imprinted genes detected among long-term shiftworkers. Environmental and molecular mutagenesis.

[152] Morini M, Astigiano S, Gitton Y, Emionite L, Mirisola V, Levi G, Barbieri O (2010). Mutually exclusive expression of DLX2 and DLX5/6 is associated with the metastatic potential of the human breast cancer cell line MDA-MB-231. BMC cancer.

[153] Sigal A, Rotter V (2000). Oncogenic mutations of the p53 tumor suppressor: the demons of the guardian of the genome. Cancer research.

[154] Shi F, Chen X, Fu A, Hansen J, Stevens R, Tjonneland A, Vogel UB, Zheng T, Zhu Y (2013). Aberrant DNA methylation of miR-219 promoter in long-term night shiftworkers. Environmental and molecular mutagenesis.

[155] Liu K, Wang R (2012). MicroRNA-mediated regulation in the mammalian circadian rhythm. Journal of theoretical biology.

[156] Liu R, Jacobs DI, Hansen J, Fu A, Stevens RG, Zhu Y (2015). Aberrant methylation of miR-34b is associated with long-term shiftwork: a potential mechanism for increased breast cancer susceptibility. Cancer causes & control : CCC.

[157] Kim IS, Baek SH (2010). Mouse models for breast cancer metastasis. Biochemical and biophysical research communications.

